# Magnetic activation of TREK1 triggers stress signalling and regulates neuronal branching in SH-SY5Y cells

**DOI:** 10.3389/fmedt.2022.981421

**Published:** 2022-12-05

**Authors:** Michael Rotherham, Yasamin Moradi, Tasmin Nahar, Dominic Mosses, Neil Telling, Alicia J. El Haj

**Affiliations:** ^1^ Healthcare Technologies Institute, School of Chemical Engineering, University of Birmingham, Heritage Building, Mindelsohn Way, Edgbaston, Birmingham, United Kingdom; ^2^ School of Pharmacy and Bioengineering, Keele University, Guy Hilton Research Centre, Stoke-on-Trent, United Kingdom; ^3^ Regenerative Medicine and Cellular Therapies, School of Pharmacy, Faculty of Science, University of Nottingham, Nottingham, United Kingdom

**Keywords:** TREK channel, magnetic nanoparticles, stress signalling pathways, mechano-stimulation, neurites

## Abstract

TWIK-related K^+^ 1 (TREK1) is a potassium channel expressed in the nervous system with multiple functions including neurotransmission and is a prime pharmacological target for neurological disorders. TREK1 gating is controlled by a wide range of external stimuli including mechanical forces. Previous work has demonstrated that TREK1 can be mechano-activated using magnetic nanoparticles (MNP) functionalised with antibodies targeted to TREK1 channels. Once the MNP are bound, external dynamic magnetic fields are used to generate forces on the TREK channel. This approach has been shown to drive cell differentiation in cells from multiple tissues. In this work we investigated the effect of MNP-mediated TREK1 mechano-activation on early stress response pathways along with the differentiation and connectivity of neuronal cells using the model neuronal cell line SH-SY5Y. Results showed that TREK1 is well expressed in SH-SY5Y and that TREK1-MNP initiate c-Myc/NF-*κ*B stress response pathways as well as Nitrite production after magnetic stimulation, indicative of the cellular response to mechanical cues. Results also showed that TREK1 mechano-activation had no overall effect on neuronal morphology or expression of the neuronal marker *β*III-Tubulin in Retinoic Acid (RA)/Brain-derived Neurotrophic factor (BDNF) differentiated SH-SY5Y but did increase neurite number. These results suggest that TREK1 is involved in cellular stress response signalling in neuronal cells, which leads to increased neurite production, but is not involved in regulating RA/BDNF mediated neuronal differentiation.

## Introduction

TREK1 is a two-pore domain outwardly rectifying potassium channel which is sensitive to a wide range of physical and chemical cues. Its main role is to maintain the resting potential of the cell and whilst highly expressed in the nervous system TREK1 is also expressed in the kidney, heart, lung and smooth muscle cells (1). TREK1 is implicated in a range of cellular and physiological processes and in the brain where it play roles in neurotransmission, pain perception and neuroprotection (2).

Stress signalling pathways mediated through transcription factors such as NF-*κ*B, c-Myc and c-FOS or the signalling molecule Nitric Oxide (NO) are established early stress response pathways to mechanical force (3–6). In the brain these signalling pathways are also intrinsically linked to neuronal physiology and development. Normal Neocortex development is dependent on c-Fos which regulates neural progenitor cell fate as well as neuritogenesis (7, 8). NF-*κ*B is known to enhance synapse growth, activity and regulate plasticity (9), whilst c-Myc regulates proliferation and survival of neural stem cells and controls the fate of neural progenitors (10). NO is also involved in neurotransmission, neuroprotection and implicated in neurite growth and synapse remodelling after nerve injury (11–13).

TREK1 is also coupled to the activity of some of these stress response pathways making this channel a potential target for regulating neuron function and fate. For instance temperature gating of TREK1 has been shown to regulate c-FOS expression (14),. Mechanical stimulation of TREK1 using MNP has been shown to upregulate NF-*κ*B gene expression in human Mesenchymal Stem Cells (hMSC) (15), whilst NO production in response to acetylcholine and bradykinin requires TREK1 in endothelial cells (16). Whilst TREK1 is gated by a plethora of different chemical and physical stimuli (1), targeted pharmacological gating of TREK1 without initiating cross-talk with other channels and receptors is difficult. Magnetic nanoparticles (MNP) have varied and established uses in biomedicine, some common examples include contrast agents, drug delivery vehicles, stem cell tracking and hyperthermia treatment of tumours (17). MNP can also be used as mechanical actuators on cell and tissues, using magnetic fields to induce a torque or pull on MNP attached to mechanosensitive channels and receptors. In this way remote stimulation leads to receptor activation, clustering, channel gating and subsequent changes in cell signalling activity and phenotype (18–21). Our previous work has shown MNP can be targeted to TREK1 and selectively activated in a variety of cell types using magnetic fields, with demonstrated applications in cell and tissue engineering (22–24).

In the current study we explored the application of our magnetic actuation system in a neural cell engineering model. We magnetically stimulated TREK1, expressed by the SH-SY5Y cell line and explored the effects of TREK1 activation on stress signalling and neuronal differentiation of SH-SY5Y cells.

## Methods

**Figure 1:**
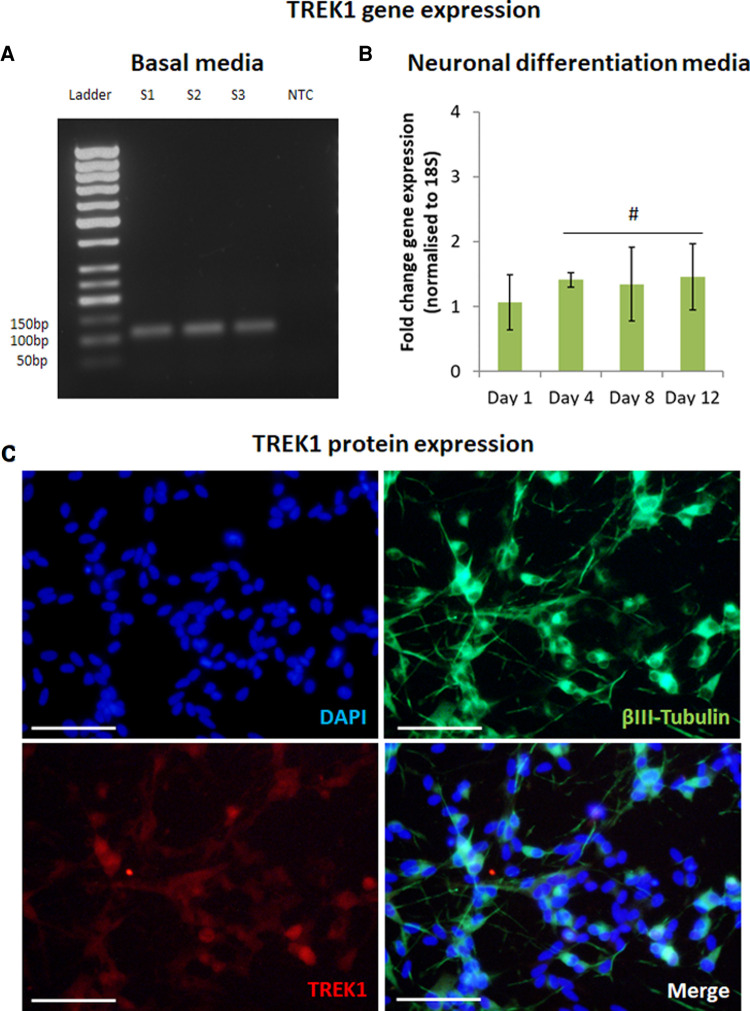
TREK1 is expressed by SH-SY5Y. Reverse transcription-PCR confirmed TREK1 gene expression in SH-SY5Y in basal media, S1-3 represent sample number (*n* = 3), NTC represents non-template control (**A**). Quantitative reverse transcription-PCR indicated TREK1 gene expression remained broadly stable with minor increases in expression over 12 days in neuronal differentiation media (**B**), *n* = 3, # represents ANOVA *p* > 0.05, bars represent mean fold change, error bars represent standard deviation. Immunofluorescent microscopy confirmed that differentiated neurons expressing *β*III-Tubulin (Green) also expressed TREK1 (Red) (**C**), Cell nuclei is shown by DAPI staining (Blue), images representative of *n* = 3, scale bar represents 50 μm.

*Cell culture.* SH-SY5Y cells (PHE, UK) were grown to confluency in basal media consisting of DMEM:F12 (Corning) supplemented with 1% antibiotics and 10% foetal bovine serum (FBS) (Gibco) and incubated at 37 ᵒC and 5% CO_2_. Culture medium was replaced twice per week, cells were passaged once they reached approximately 80% confluency. Cells of passage 18–30 were used in all experiments. For neuronal differentiation, cells were seeded on pre-coated laminin (Sigma) plates and cultured in basal media for 24 h to allow cell attachment. Cells were differentiated using a neuronal differentiation protocol based on Encinas et al. (25). Briefly, media was changed to neuronal differentiation media consisting of basal media with the addition of 10 mM Retinoic Acid (RA) (Sigma) for 4 days after which media was switched to serum free media with RA and 25 ng/ml Brain Derived Neurotrophic Factor (BDNF) (R&D systems) for a minimum of 8 days. Neuronal differentiation was confirmed microscopically by observing morphology changes and staining for betaIII-Tubulin (Figure 1C).

*MNP functionalisation.* 250 nm SPIO carboxyl functionalised magnetic nanoparticles (Micromod, Germany) were covalently coated with anti-TREK1 antibody (Alomone labs) by carbodiimide activation method as described previously (26). 2 mg of particle suspension was activated with EDAC/NHS in 0.5M MES buffer (Sigma) for 1 h at room temperature. After washing on a permanent magnet and re-suspension in 0.1M MES buffer (Sigma), particles were coated overnight at 4 °C with 40 µg of either anti-Rabbit-IgG (Abcam) or anti-Rabbit IgG-594 in 0.1M MES buffer. The coating solution was aspirated on a permanent magnet and MNP re-suspended in 0.1M MES buffer containing 20 µg anti-TREK1 and mixed for 3 h at room temperature. The particle suspension was then blocked with 25 mM Glycine (Sigma), washed in PBS (Lonza) and re-suspended in 2 ml PBS (Fisher) and stored at 4 °C.

*Cell MNP labelling.* Adherent SH-SY5Y cells were cultured in reduced serum basal media for 3 h prior to addition of 25 µg MNP/2 × 10^5^ cells. Cells were incubated with MNP for 1.5 h, then washed with PBS to remove unbound particles before addition of fresh media. For the neuronal differentiation study cells were cultured in neuronal differentiation media for 4 days before labelling with MNP and washing as above. Media was then changed to serum free media with RA and BDNF and MNP and cultured for a minimum of 8 days.

*Magnetic stimulation.* Magnetically stimulated groups were treated with ≥25 mT magnetic fields provided by arrays of NdFeB magnets in 1 h sessions at 1 Hz using a vertically oscillating magnetic force bioreactor (MFB), (MICA Biosystems, UK). The bioreactor was housed in a cell culture incubator maintained at 37 °C, 5% CO_2_. Non-stimulated control groups were cultured under identical conditions (without magnetic field).

*Polymerase Chain Reaction (PCR)*. At each time-point, total RNA was extracted using an RNAeasy extraction kit (Qiagen) according to the manufacturer's instructions. Reverse transcription was performed on 1 *μ*g RNA using a high-capacity reverse transcription kit (Applied Biosystems). PCR reaction mixes were prepared using diluted cDNA mixed with PCR master mix (Applied Biosystems) and commercially available primers for KCNK2 (TREK1), NF-*κ*B, c-Myc, c-FOS, NOS3 and 18 s (Qiagen). Thermocycling was performed on a Stratagene MX3000P system. Gene expression was normalised to 18 s. Fold change in gene of interest expression was calculated using the delta-delta CT method. PCR products were resolved on a 2% Agarose gel and imaged using a GelDoc-It2 imager system (UvP).

*Immunocytochemistry.* For establishment of neuronal differentiation and TREK1 expression, cells were fixed with 4% PFA in PBS (Fisher) for 15mins, permeabilised with 0.1% Triton-X in PBS for 10 min, washed with PBS, then blocked with 2% BSA/PBS for 2 h. Cells were then stained with *β*3-tubulin (Abcam) diluted 1:1000 and TREK1 (Alomone labs) diluted 1:100. All primary antibodies were diluted in 1% BSA/PBS and incubated overnight at 4 °C. After washing with PBS, cells were stained with goat anti-mouse-FITC (Sigma) diluted 1:1000 and Rabbit anti-goat-555 (Life technologies) diluted 1:2000 for TREK1. Both secondary antibodies were diluted in 1% BSA/PBS and incubated for 2 h at room temperature. Cells were washed with PBS X3 cells then counterstained with DAPI (Sigma) for 15 min and stored in PBS. Fluorescence microscopy was performed on an Eclipse Ti-S microscope (Nikon).

MNP labelling of cells was determined using immunofluorescence to identify the dextran matrix of the MNP. After labelling with MNP cells were fixed, blocked, then stained with an anti-dextran antibody (Stem Cell Technologies) diluted 1:1000 in 1% BSA in PBS overnight at 4 °C. Cells were then washed with PBS and stained with anti-mouse-FITC secondary antibody (Sigma) diluted 1:1000 in 1% BSA in PBS for 1 h at room temperature. Cells were then washed with PBS and counterstained with DAPI (Sigma) to visualise cell nuclei and Phalloidin-iFluor-594 (Stratech) to visualise F-Actin.

MNP binding to TREK1 channels was determined using immunofluorescence to stain TREK1 channels along with MNP fluorescence from anti-Rabbit-IgG-594 coated MNP. Cells were cultured on glass coverslips before labelling with MNP as above. For the TREK-blocked groups TREK-MNP were incubated with TREK1 blocking peptide for 2 h at room temperature (Alomone labs), at a concentration of 1*μ*g peptide per μg of anti-TREK coated on the MNP. After MNP labelling, cells were fixed, permeabilised, then blocked as above. Samples were then stained with an anti-TREK1 antibody raised against the C-terminus of the TREK1 channel (Santa Cruz) which was diluted 1:200 in 1% BSA in PBS and incubated overnight at 4 °C. Cells were then washed with PBS then stained with anti-mouse-ATTO 488 sary antibody (Sigma) diluted 1:1000 in 1% BSA in PBS for 1 h at room temperature. Cells were then washed with PBS and counterstained with DAPI (Sigma) to visualise cell nuclei before storing in PBS. Coverslips were mounted onto slides (Fisher) using Permaflour (Fisher). Fluorescence microscopy was performed on an EVOS M5000 microscope (ThermoFisher).

*Griess assay.* At each time-point media was aspirated, cells washed with PBS, then lysed using 0.1% Triton-X in d.H_2_O, samples were then stored at −20 °C before analysis. Nitrite concentration in cell lysates was quantified using the Griess assay (Fisher) according to the manufacturer's instructions. Values were normalised to the DNA content of the respective cell lysates using a PicoGreen assay (Invitrogen) according to the manufacturer's instructions.

*Image analysis.* The mean gray value of BIII-Tubulin and DAPI staining was quantified using ImageJ, BIII-Tubulin staining intensity was then normalised to DAPI staining. Neuritogenesis was quantified from BIII-Tubulin stained samples using the Neurite Analyser ImageJ plug-in developed by Haas et al. (27).

*Statistical analysis.* All data is presented as means +/- Standard Deviation unless stated otherwise. Statistical significance at 95% confidence level was determined using 1-way or 2-way ANOVA with post-hoc Tukey tests using Mini-tab (v16). Data was tested for normality and equal variance before analysis. For non-normal/unequal variance data sets, non-parametric Kruskal-Wallis and post-hoc Dunn's test were performed, with statistical significance at 95% confidence level. Analysis was performed using Graphpad Prism 6 or PlotsOfDifferences web application (28).

## Results

### TREK1 is expressed by differentiated SH-SY5Y cells

The expression of TREK1 was first confirmed in SH-SY5Y cells cultured in basal media using rt-PCR (Figure 1A). SH-SY5Y were differentiated using a combination of RA and Brain Derived Neurotrophic Factor (BDNF) over 12 days with q-rt-PCR used to track changes in TREK1 gene expression during the differentiation process. TREK1 expression was found to be marginally increased by 1.5-fold (not statistically significant) during neuronal differentiation (Figure 1B). Immunocytochemistry was then used to assess neuronal differentiation and TREK1 expression in differentiated SH-SY5Y cells. *β*III tubulin staining confirmed neuronal differentiation with almost all *β*III Tubulin positive cells also co-expressing TREK1, mainly at the cell bodies (Figure 1C).

### SH-SY5Y tagging with MNP

**Figure 2:**
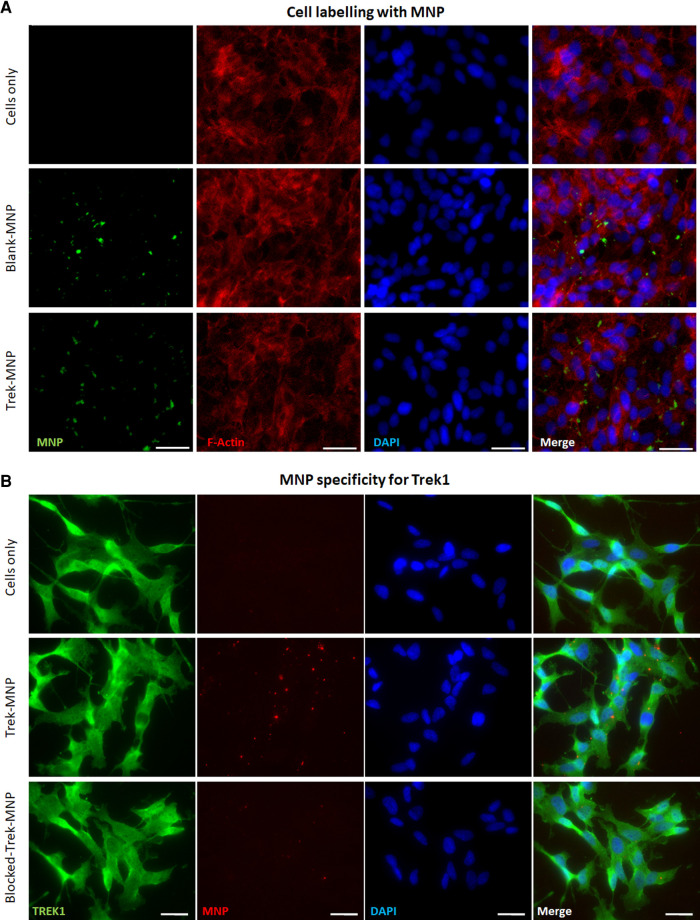
TREK-MNP labelling of SH-SY5Y. (**A**) Immunofluorescent microscopy showing unlabelled cells (top row) and cells labelled with Blank-MNP (middle row) or TREK-MNP (bottom row). MNP are shown by Dextran staining (green), cell cytoskeleton is shown by Actin staining (red) and cell nuclei are shown by DAPI staining (Blue). Images representative of *n* = 3, scale bar represents 25 μm. (**B**) Immunofluorescent microscopy showing unlabelled cells (top row), cells labelled with fluorescent TREK-MNP (middle row) or fluorescent TREK-MNP pre-blocked with a TREK blocking peptide (bottom row). TREK1 channels are shown in green, MNP are shown in red and cell nuclei are shown by DAPI staining (Blue). Images representative of *n* = 3, scale bar represents 50 μm.

Dextran-MNP with a diameter 250 nm were functionalised with anti-TREK1 antibodies, then used to tag SH-SY5Y cells. Immunocytochemistry was used to stain and visualise the dextran matrix of the MNP coating and to qualitatively assess MNP association with the cells. Fluorescent microscopy confirmed both Control (Blank-MNP) and TREK-MNP binding to cells post labelling (Figure 2A). Quantification of bound MNP indicated an average of 2–3 MNP clusters bound per cell (Supplementary Figure S1). Immunocytochemistry was also used to determine the specificity of the TREK-MNP for TREK1 channels. Cells were immunostained for TREK1 after labelling with TREK-MNP that had been pre-coated with a fluorescent secondary antibody, to allow visualisation of the particles (Figure 2B). Results confirmed labelling of TREK1 channels with the TREK-MNP (middle row), whilst pre-incubation of the TREK-MNP with a TREK blocking peptide resulted in reduced binding to the cells (bottom row). Unlabelled cells are shown in the top row.

### TREK1-MNP activate stress-response gene expression

**Figure 3:**
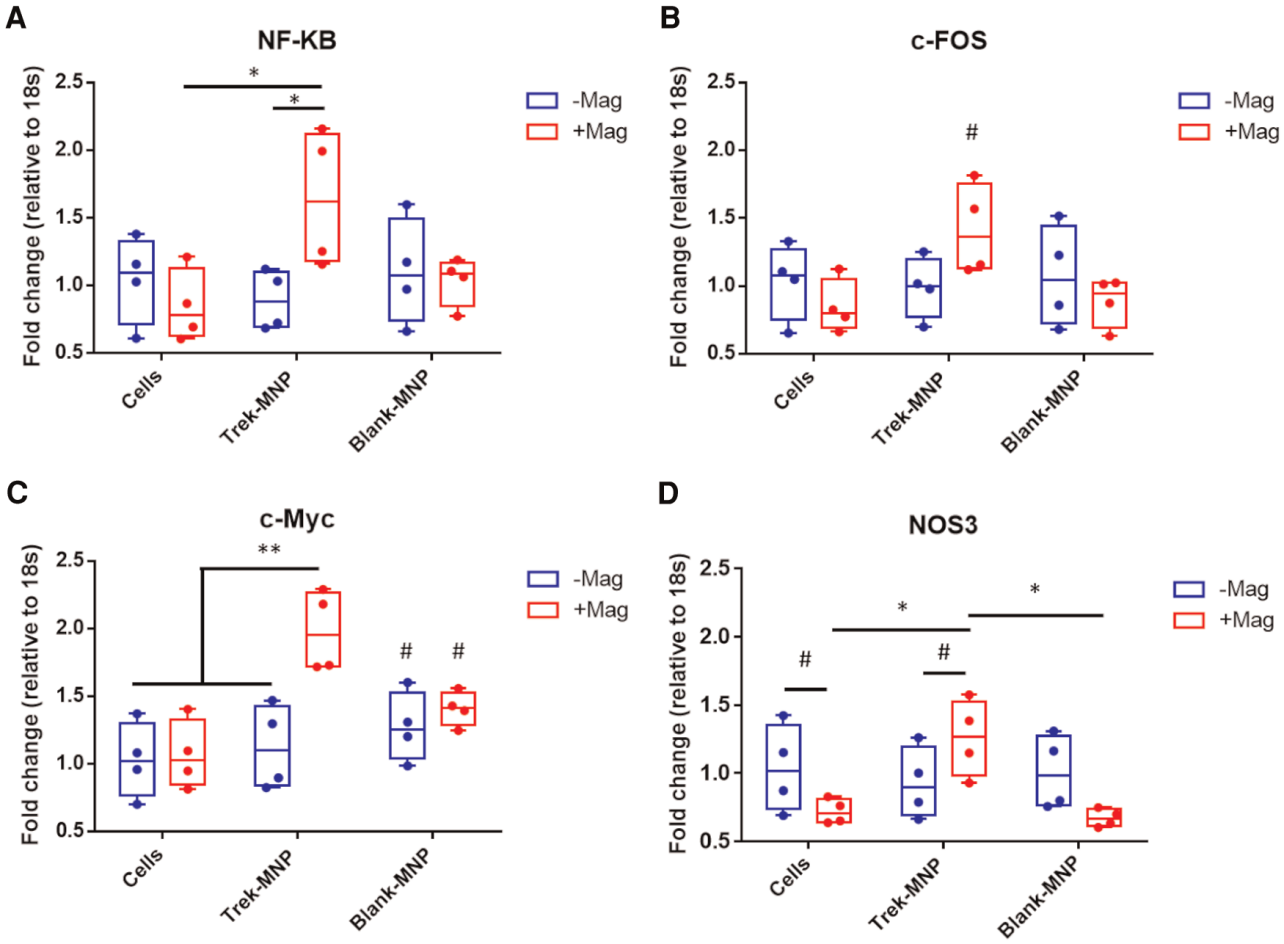
TREK-MNP induce stress-response gene expression in SH-SY5Y. Quantitative-reverse transcription-PCR was used to measure NF-*κ*B, c-Fos, c-Myc and NOS3 gene expression 24 h after treatment with MNP and or magnetic field stimulation. Treatment with TREK-MNP and magnetic field stimulation resulted in elevated NF-*κ*B expression compared to cells + magnet control group and Trek-MNP alone treatment. In contrast treatment with control (Blank)-MNP had no significant effect on NF-*κ*B expression (**A**). The same overall trend was also observed across the groups with c-Fos gene expression (not statistically significant), (**B**). TREK-MNP and magnetic field stimulation also resulted in significantly elevated c-Myc expression vs. controls, whist control (Blank)-MNP caused no significant changes in c-Myc expression (**C**). NOS3 gene expression was significantly elevated after treatment with Trek-MNP + magnetic field stimulation compared to the magnet control group (Cells + magnet) and Blank-MNP + magnet control group. In contrast treatment with control (Blank)-MNP with or without magnetic field stimulation had no effect on NOS3 expression compared to the respective cell only controls (**D**). *n* = 4, plots represent median fold change with IQR, * represents *p* < 0.05, # represents *p* > 0.05 (2-way ANOVA).

The role of TREK1 in triggering stress response pathways was explored by assessment of known early stress-response genes using q-rt-PCR (Figure 3). NF-*κ*B gene expression was significantly elevated in SH-SY5Y 24 h after a 1 h treatment with TREK-MNP and magnetic stimulation. In contrast treatment with TREK-MNP without magnetic stimulation or treatment with control (blank-MNP) with or without magnetic field stimulation had no clear effect on NF-*κ*B expression (Figure 3A). Expression of c-FOS was also elevated (but did not reach statistical significance) in response to treatment with TREK-MNP in combination with magnetic field stimulation, whilst expression across all other groups remained around the baseline (Figure 3B). Expression of c-Myc was again significantly elevated when cells were treated with TREK-MNP in conjunction with magnetic field stimulation, whilst treatment with TREK-MNP alone or control (blank-MNP) had modest (non-significant) effects on c-Myc expression (Figure 3C). NOS3 expression was also significantly elevated by TREK-MNP and magnetic field stimulation compared to both magnet control groups (cells + magnet and blank-MNP + magnet), whilst treatment with Control (blank-MNP) with or without magnetic field stimulation had no overall effect on expression compared to the respective cells only control groups (Figure 3D).

### TREK1 stimulation initiates nitrite production

**Figure 4:**
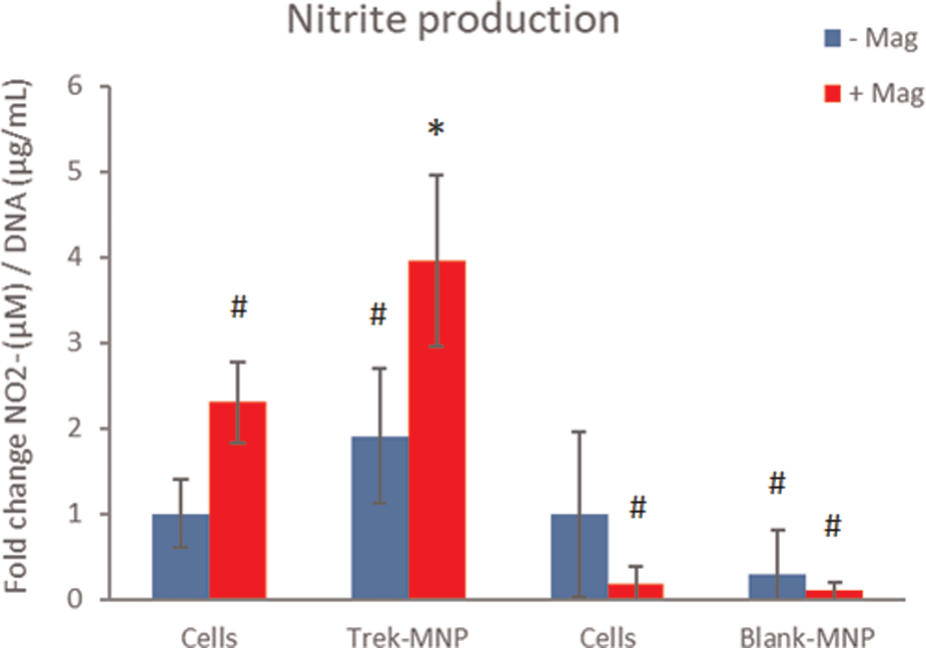
TREK-MNP induce NO signalling in SH-SY5Y. Nitrite prduction was assayed using the Greiss assay. Treatment with TREK-MNP plus magnetic field stimulation resulted in a 4-fold increase in Nitrite production whilst uncoated (Blank-MNP) had no effect on Nitrite production. *n* = 3-4, bars represent mean fold change, * represents *p* < 0.05, # represents *p* > 0.05 (2-way ANOVA).

Nitrite (NO^−^_2_) production is a proxy for NO production by cells and another stress-response indicator. NO^−^_2_ production by SH-SY5Y cells in response to MNP and magnetic stimulation was assessed using the Griess assay, a routinely used assay for NO^−^_2_. Treatment of SH-SY5Y cells with Trek-MNP and magnetic field stimulation led to a significant four-fold increase in NO^−^_2_ production 24 h post-treatment. NO^−^_2_ production was also elevated by magnetic stimulation alone and Trek-MNP alone but did not reach statistical significance (Figure 4). Treatment with Control (Blank-MNP) marginally reduced NO^−^_2_ production compared to its experimental control (not statistically significant). The DNA content of the cell lysates was also assessed and used as a normalisation factor for NO^−^_2_ production in order to control for cell number. Treatment with TREK-MNP or control (blank-MNP), with or without magnetic field stimulation, caused negligible (non significant) changes in DNA content (Supplementary Figure S2).

### TREK1 does not regulate neuronal differentiation

The effect of TREK-targeted MNP on neuronal differentiation was then evaluated using immunocytochemistry to monitor neuronal morphology and expression of the pan-neuronal marker BIII-Tubulin after TREK stimulation. Cells were cultured in reduced serum neuronal induction media (RA media) for four days then labelled with MNP. Media was then switched to neuronal induction media containing RA and BDNF for a further 8 days with intermittent magnetic stimulation.

**Figure 5:**
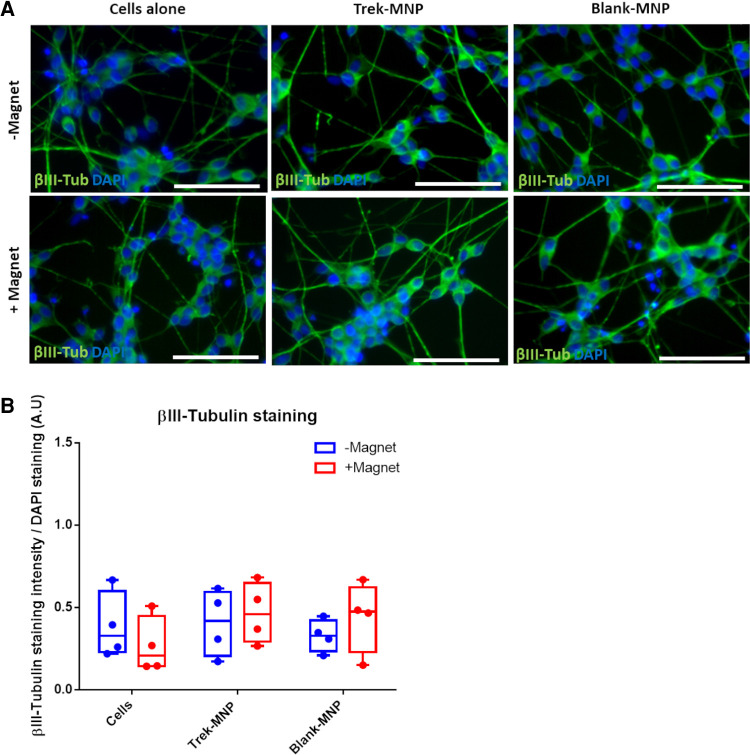
TREK-MNP do not regulate SH-SY5Y neuronal differentiation. Immunofluorescent microscopy showed that TREK-MNP (alone or with magnetic field stimulation) do not influence neuron morphology or expression of *β*III-tubulin during RA/BDNF mediated neuronal differentiation (**A**). *β*III-Tubulin is shown in green, cell nuclei are shown by DAPI staining (Blue), images representative of *n* = 4, bar represents 50 μm. Pixel intensity analysis of *β*III-Tubulin staining confirmed that neither TREK-MNP nor Blank-MNP had any significant effect on *β*III-Tubulin staining intensity (**B**). *n* = 4, plots represent median fold change and IQR.

Cells appeared to differentiate normally after treatment with TREK- or Control-MNP (with or without magnetic field stimulation) with cells displaying a typical neuronal morphology with extended processes. Cells also strongly expressed *β*III-Tubulin regardless of MNP or magnetic field treatment (Figure 5A). Quantification of *β*III-Tubulin staining confirmed that both TREK- and the Control (Blank-MNP) maintained *β*III-Tubulin expression with neither treatment having any significant effects overall on staining intensity (Figure 5B).

### TREK1 regulates neuritogenesis during neuronal differentiation

**Figure 6:**
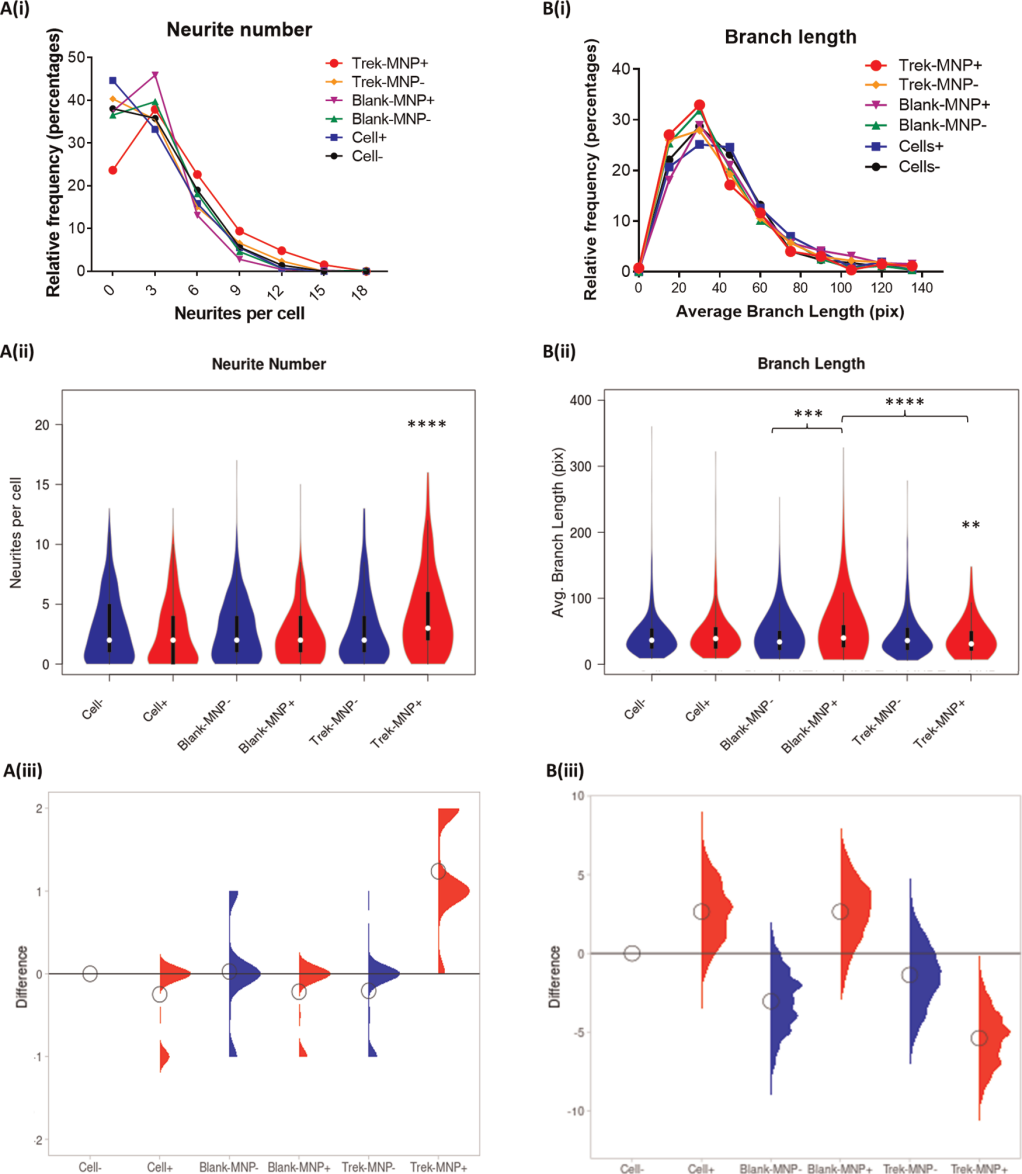
TREK-MNP increase neurite number and decrease branch length. Neurite analysis of Immunofluorescent images indicated that TREK-MNP with magnetic field stimulation significantly increased the overall number of neurites per cell as shown by histogram (**A(i)**) and violin plots (**A(ii)**) whilst all other groups had no significant effect and remained similar to the control (cells only). The differences between the groups relative to the cells only control are shown by the median difference plots (**A(iii)**). Branch length analysis of neurites indicated that TREK-MNP (with magnetic field stimulation) significantly decreased neurite branch length compared to the respective controls (cells + magnet and Blank-MNP + Magnet) as shown by histogram (**B(i)**) and violin plots (**B(ii)**). Blank-MNP + magnet marginally increased neurite branch length compared to Blank-MNP alone or TREK-MNP. The differences between the groups are shown by the median difference plots (**B(iii)**). Histograms and Violin plots represent median value (median denoted by white circles); box limits indicate the 25th and 75th percentiles; polygons represent density estimates of data and extend to extreme values. Differences between groups were compared using the non-parametric Kruskal-Wallis test, with Dunn's post-hoc multiple comparison. A minimum of 271 cells were analysed for each group.

The effect of TREK1 stimulation on neuritogenesis was also assessed by quantifying Neurite number, branch number, branch length and number of neurite junctions using an ImageJ analysis plugin NeuriteAnalyser. Results showed that TREK-MNP with magnetic field stimulation led to an overall significant increase in neurite number per cell (median of three neurites/cell) compared to control groups (median of two neurites/cell), whilst control (Blank)-MNP had no effect on neurite number (Figure 6A(i–iii)). In contrast, the average neurite branch length was significantly decreased in response to TREK-MNP with magnetic field stimulation, whilst Control (Blank-MNP) with magnetic field stimulation slightly increased branch length compared to Blank-MNP alone or the TREK-MNP treated groups, (Figure 6B(i–iii)). The number of branches per neurite (Supplementary Figure S3A) and number of neurite junctions (Supplementary Figure S3B) remained consistent across all groups with no overall effect observed.

## Discussion

The TREK1 potassium channel plays a range of roles in the nervous system, including neurotransmission and pain perception. The channel is gated by a wide range of physical and chemical stimuli, modulators of TREK1 are therefore of interest for pharmaceutical drug discovery (1, 2). MNP represent a novel method for remotely controlling ion channel activity using magnetic fields and this study adds to the growing area of MNP mediated mechano-activation of cells to trigger intra-cellular signalling pathways to control cell behaviour (29). In this work we explored the effects of MNP-mediated mechano-activation of the TREK1 potassium channel expressed by SH-SY5Y cells to influence stress signalling, neuronal differentiation and neuronal connectivity.

External stress factors such as heat, nutrient starvation or UV light have been shown to alter neuron morphology and induce neurite growth (30). Mechanical forces are another form of external stress and control transcription of early stress response genes, which can be used as early indicators of cellular mechanical stress (3–5, 31). Mechanical forces such as stretch are also known to influence neurite extension, even in the absence of neurogenic factors such as RA (32). Tensile forces provided by MNP have also been shown to promote neurite elongation in PC12 cells (33). This demonstrates the beneficial role that mechanical forces can play in neural tissue engineering.

In the brain, stress response pathways are activated after injury and are involved in initiating repair. For instance, c-Myc is known to be up-regulated after nerve injury and its expression was shown to drive transcription of a range of regeneration associated genes (34). Our results show that targeted magnetic activation of TREK1 using MNP leads to increased expression of common stress response genes, including c-Myc, which is consistent with activation of cellular stress response pathways in response to mechanical stimulation of cells.

We also observed some fluctuations in the expression of these stress response genes in response to non-specific (blank)-MNP with and without magnetic fields (e.g., c-Myc and NF-*κ*B). This response may be expected given that these MNP still bind to the cells in a non-specific manner and in similar numbers to TREK targeted MNP. Whilst the cell-binding affinity of control-MNP is likely to be different compared to antibody binding *via* Trek-MNP, the fact that control-MNP still bind to cells suggests that they are capable of transducing generic force to the cell membrane. In principle this could trigger expression of the same stress response genes under certain conditions. Previous work from our group has shown that control MNP do not activate downstream signalling pathways in the same manner as antibody targeted MNP. However, the cell binding properties and signalling activity of various control-MNP (either uncoated or non-specifically targeted) can be variable depending on the cell type, experimental conditions and assay used (18, 19, 21, 35). A major factor in determining the cell binding properties of nanomaterials is the protein corona which forms when materials are immersed in biological fluids. The composition of proteins in the corona may differ depending on whether the MNP are uncoated or pre-functionalised with biological ligands, which may in turn affect the cell binding efficacy. This factor must therefore be carefully considered in experiments using serum containing conditions, due to the effect that the corona has on regulating MNP-cell association and uptake (36, 37). It is also plausible that the control-MNP are binding to glycan-binding or scavenger receptors through interaction with the dextran matrix of the MNP (38, 39). Altogether this highlights the importance of carefully selecting the appropriate control coatings on the surface of MNP and considering the effect of the protein corona when examining the cellular responses to targeted MNP.

NF-*κ*B signalling is a ubiquitous stress response pathway and is expressed in most cells. We have previously demonstrated NF-*κ*B upregulation in response to MNP mediated TREK1 activation in hMSC (15), the fact this effect was also observed in the current study suggests that TREK1 is a regulator of NF-*κ*B signalling across multiple cells from varied tissues. The gating mechanism of TREK1 has been implicated in differentially regulating stress response gene expression. For example, temperature gating of TREK1 has been linked to c-FOS expression. In one study cold temperatures resulted in TREK1 closing and increased c-FOS expression but at the same time TREK1 knockout also reduced overall c-FOS expression (14), whilst in our study c-FOS was mildly elevated. This suggests that the functional effects of TREK1 activation can vary depending on the cell type and/or the type of stimulus received.

We also examined the production of reactive oxygen species after magnetic Trek activation. Nitrite is a stable reaction metabolite of NO, a free radical with an extremely short half-life in the oxidising environment of cells (40). NO has a number of physiological roles in the central nervous system which includes neurotransmission (11) and the brain's response to injury, ischaemia or stroke where eNOS derived NO exhibits neuroprotective effects by increasing blood flow to promote brain repair (13). NO has also been shown to increase neuronal growth and promote synaptic remodelling (12). The link between NO and TREK1 has been made before; NO is thought to activate potassium channels, as well as regulate TREK1 activity (41, 42). Another study has shown that NO production in endothelial cells requires TREK1 activity (16), in related studies TREK1 was thought to induce calcium flux and increase NO synthesis in endothelial cells (43–45). Our results showed that TREK1 activation led to increased NOS3 gene expression and Nitrite production, whilst this response was largely absent when cells were treated with control MNP. This confirms the link between TREK1 and NO production and is consistent with the cellular stress response to mechanical activation (6). Altogether, this suggests a role for TREK channels in regulating the cellular response to mechanical loading *via* NO signalling.

We then explored the effects of magnetic TREK1 activation on neuronal differentiation. We intermittently stimulated TREK1 expressed by SH-SY5Y cells cultured in neuronal differentiation media. Our results indicated that TREK1 stimulation had no clear effect on neuronal differentiation and did not appear to regulate the differentiation process. This is unsurprising given that TREK1 appears to play its main roles in mature neuron function and neurotransmission rather than neural progenitor cell differentiation (2). In our previous work we demonstrated that activation of the Wnt pathway augmented dopaminergic marker expression in differentiated SH-SY5Y cells (19). This indicates that receptor selection is important when considering targets for stimulating optimal neuronal differentiation using MNP. Perhaps just as importantly, the differentiating SH-SY5Y cells in this study appeared to tolerate MNP-mediated TREK1 stimulation, which had no obvious adverse effect on neuronal differentiation capacity or cell morphology. This is an important factor to consider if MNP based therapies are to be adopted for neuronal tissue engineering.

Our results did however indicate that control-MNP and magnetic fields influenced neurite branch length to some extent. Interestingly, similar results have been observed in other studies utilising non-specifically targeted or internalised MNP. In these studies magnetic fields were used to enhance neurite extension and exert directional control over neurite direction (33, 46, 47). This suggests a role for non-specific MNP in promoting directional neurite extension under applied magnetic fields. In contrast to non-specifically bound MNP, targeted TREK1 stimulation resulted in increased neuritogenesis. In our system MNP-mediated TREK1 activation caused an overall increase in neurite production and decrease in neurite branch length. These effects can be explained by TREK1′s links with stress response and repair pathway activation. Given the fact that stress mediators play roles in regulating neuronal connectivity, neuritogenesis and synaptogenesis (8, 9, 12), it is reasonable to suggest that increased neurite production in our system is a result of increased stress signalling and NO production after mechano-activation of TREK1. An increase in neurite number would subsequently allow decreased branch length as neuron connectivity may be achieved by increased neurite numbers alone.

## Conclusions

New tools are required to regulate cell behaviour in the growing cell therapy industry. MNP are versatile materials within the tissue engineering toolkit and have growing applications in regenerative medicine including the regulation of cell signalling, function and fate.

Our results have shown that magnetic activation of TREK1 potassium channel initiates stress-response gene expression and nitrite production in neuronal SH-SY5Y cells, consistent with the cellular response to mechanical loading. TREK1 stimulation did not appear to influence neuronal differentiation of SH-SY5Y cells but did increase neuritogenesis, suggesting TREK1 may play a role in regulating neuronal connectivity. The approach presented here has useful applications for regulating neuronal stress signalling and network connectivity which could form part of future injectable cell therapies for neuronal tissue engineering, or treatment of neurodegenerative diseases which require restoration of neuronal connectivity.

## Data Availability

The raw data supporting the conclusions of this article will be made available by the authors, without undue reservation.

## References

[1] Djillani A Mazella J Heurteaux C Borsotto M. Role of TREK-1 in health and disease, focus on the central nervous system. Front Pharmacol. (2019) 10(379). 10.3389/fphar.2019.00379 31031627 PMC6470294

[2] Honoré E. The neuronal background K2P channels: focus on TREK1. Nat Rev Neurosci. (2007) 8(4):251–61. 10.1038/nrn2117 17375039

[3] Peake MA Cooling LM Magnay JL Thomas PBM Haj AJE. Selected contribution: regulatory pathways involved in mechanical induction of c-fos gene expression in bone cells. J Appl Physiol. (2000) 89(6):2498–507. 10.1152/jappl.2000.89.6.2498 11090608

[4] Perera PM Wypasek E Madhavan S Rath-Deschner B Liu J Nam J Mechanical signals control SOX-9, VEGF, and c-mycexpression and cell proliferation during inflammation via integrin-linked kinase, B-raf, and ERK1/2-dependent signaling in articular chondrocytes. Arthritis Res Ther. (2010) 12(3):R106. 10.1186/ar3039 20509944 PMC2911896

[5] Novack DV. Role of NF-*κ*B in the skeleton. Cell Res. (2011) 21(1):169–82. 10.1038/cr.2010.159 21079651 PMC3193402

[6] Harrison DG Widder J Grumbach I Chen W Weber M Searles C. Endothelial mechanotransduction, nitric oxide and vascular inflammation. J Intern Med. (2006) 259(4):351–63. 10.1111/j.1365-2796.2006.01621.x 16594903

[7] Velazquez FN Prucca CG Etienne O Astolfo DS Silvestre DC Boussin FD Brain Development is Impaired in c-fos -/- Mice. Oncotarget. (2015) 6:16883–901. 10.18632/oncotarget.4527. 26143639 PMC4621926

[8] Gil GA Bussolino DF Portal MM Alfonso Pecchio A Renner ML Borioli GA c-Fos activated phospholipid synthesis is required for neurite elongation in differentiating PC12 cells. Mol Biol Cell. (2004) 15(4):1881–94. 10.1091/mbc.e03-09-0705 14767061 PMC379284

[9] Dresselhaus EC Meffert MK. Cellular specificity of NF-*κ*B function in the nervous system. Front Immunol. (2019) 10(1043). 10.3389/fimmu.2019.01043 31143184 PMC6520659

[10] Zaytseva O Kim N-h Quinn LM. MYC In brain development and cancer. Int J Mol Sci. (2020) 21(20):7742. 10.3390/ijms21207742 33092025 PMC7588885

[11] Esplugues JV. NO As a signalling molecule in the nervous system. Br J Pharmacol. (2002) 135(5):1079–95. 10.1038/sj.bjp.0704569 11877313 PMC1573233

[12] Cooke RM Mistry R Challiss RAJ Straub VA. Nitric oxide synthesis and cGMP production is important for neurite growth and synapse remodeling after axotomy. J Neurosci. (2013) 33(13):5626–37. 10.1523/JNEUROSCI.3659-12.2013 23536077 PMC6705058

[13] Garry PS Ezra M Rowland MJ Westbrook J Pattinson KTS. The role of the nitric oxide pathway in brain injury and its treatment — from bench to bedside. Exp Neurol. (2015) 263:235–43. 10.1016/j.expneurol.2014.10.017 25447937

[14] Stebe S Schellig K Lesage F Breer H Fleischer J. The thermosensitive potassium channel TREK-1 contributes to coolness-evoked responses of grueneberg ganglion neurons. Cell Mol Neurobiol. (2014) 34(1):113–22. 10.1007/s10571-013-9992-x 24101433 PMC11488964

[15] Rotherham M El Haj AJ. Remote activation of the wnt/beta-catenin signalling pathway using functionalised magnetic particles. PloS one. (2015) 10(3):e0121761. 10.1371/journal.pone.0121761 25781466 PMC4363733

[16] Garry A Fromy B Blondeau N Henrion D Brau F Gounon P Altered acetylcholine, bradykinin and cutaneous pressure-induced vasodilation in mice lacking the TREK1 potassium channel: the endothelial link. EMBO Rep. (2007) 8(4):354–9. 10.1038/sj.embor.7400916 17347672 PMC1852759

[17] Bárcena C Sra AK Gao J. Applications of magnetic nanoparticles in biomedicine. In: Liu JP Fullerton E Gutfleisch O Sellmyer DJ, editors. Nanoscale magnetic materials and applications. Boston, MA: Springer US (2009). p. 591–626.

[18] Hu B Rotherham M Farrow N Roach P Dobson J El Haj AJ. Immobilization of wnt fragment peptides on magnetic nanoparticles or synthetic surfaces regulate wnt signaling kinetics. Int J Mol Sci. (2022) 23(17):10164. 10.3390/ijms231710164 36077561 PMC9456016

[19] Rotherham M Nahar T Goodman T Telling N Gates M El Haj A. Magnetic mechanoactivation of wnt signaling augments dopaminergic differentiation of neuronal cells. Adv Biosyst. (2019) 3(9):1900091. 10.1002/adbi.201900091 32648650

[20] Hu B El Haj AJ Dobson J. Receptor-targeted, magneto-mechanical stimulation of osteogenic differentiation of human bone marrow-derived mesenchymal stem cells. Int J Mol Sci. (2013) 14(9):19276–93. 10.3390/ijms140919276 24065106 PMC3794833

[21] Gonçalves AI Rotherham M Markides H Rodrigues MT Reis RL Gomes ME Triggering the activation of activin A type II receptor in human adipose stem cells towards tenogenic commitment using mechanomagnetic stimulation. Nanomed: Nanotechnol Biol and Med. (2018) 14(4):1149–59. 10.1016/j.nano.2018.02.008 29471171

[22] Hughes S McBain S Dobson J El Haj AJ. Selective activation of mechanosensitive ion channels using magnetic particles. J R Soc Interface. (2008) 5(25):855–63. 10.1098/rsif.2007.1274 18077244 PMC2495030

[23] Markides H McLaren JS Telling ND Alom N Al-Mutheffer E Oreffo ROC Translation of remote control regenerative technologies for bone repair. npj Regen Med. (2018) 3(1):9. 10.1038/s41536-018-0048-1 29675269 PMC5904134

[24] Unnithan AR Sasikala ARK Shrestha BK Lincoln A Thomson T El Haj AJ. Remotely Actuated Magnetic Nanocarpets for Bone Tissue Engineering: Non-Invasive Modulation of Mechanosensitive Ion Channels for Enhanced Osteogenesis.n/a(n/a):2201311.

[25] Encinas M Iglesias M Liu Y Wang H Muhaisen A Ceña V Sequential treatment of SH-SY5Y cells with retinoic acid and brain-derived neurotrophic factor gives rise to fully differentiated, neurotrophic factor-dependent, human neuron-like cells. J Neurochem. (2000) 75(3):991–1003. 10.1046/j.1471-4159.2000.0750991.x 10936180

[26] Henstock JR Rotherham M El Haj AJ. Magnetic ion channel activation of TREK1 in human mesenchymal stem cells using nanoparticles promotes osteogenesis in surrounding cells. J Tissue Eng. (2018) 9:2041731418808695. 10.1177/2041731418808695 30397432 PMC6207961

[27] Haas AJ Prigent S Dutertre S Le Dréan Y Le Page Y. Neurite analyzer: an original Fiji plugin for quantification of neuritogenesis in two-dimensional images. J Neurosci Methods. (2016) 271:86–91. 10.1016/j.jneumeth.2016.07.011 27450924

[28] Goedhart J. Plotsofdifferences – a web app for the quantitative comparison of unpaired data. bioRxiv. (2019):578575. 10.1101/578575

[29] Rotherham M Nahar T Broomhall TJ Telling ND El Haj AJ. Remote magnetic actuation of cell signalling for tissue engineering. Curr Opin Biomed Eng. (2022) 24:100410. 10.1016/j.cobme.2022.100410

[30] Hart MP. Stress-Induced neuron remodeling reveals differential interplay between neurexin and environmental factors in caenorhabditis elegans. Genetics. (2019) 213(4):1415–30. 10.1534/genetics.119.302415 31558583 PMC6893388

[31] Ziegler T Silacci P Harrison VJ Hayoz D. Nitric oxide synthase expression in endothelial cells exposed to mechanical forces. Hypertension. (1998) 32(2):351–5. 10.1161/01.HYP.32.2.351 9719066

[32] Higgins S Lee JS Ha L Lim JY. Inducing neurite outgrowth by mechanical cell stretch. Biores Open Access. (2013) 2(3):212–6. 10.1089/biores.2013.0008 23741633 PMC3666214

[33] Raffa V Falcone F De Vincentiis S Falconieri A Calatayud MP Goya GF Piconewton mechanical forces promote neurite growth. Biophys J. (2018) 115(10):2026–33. 10.1016/j.bpj.2018.10.009 30473016 PMC6303536

[34] Shin HY Kwon MJ Lee EM Kim K Oh YJ Kim HS Role of <em > myc</em > proto-oncogene as a transcriptional hub to regulate the expression of regeneration-associated genes following preconditioning peripheral nerve injury. J Neurosci. (2021) 41(3):446–60. 10.1523/JNEUROSCI.1745-20.2020 33262248 PMC7821863

[35] Henstock JR Rotherham M Rashidi H Shakesheff KM El Haj AJ. Remotely activated mechanotransduction via magnetic nanoparticles promotes mineralization synergistically with bone morphogenetic protein 2: applications for injectable cell therapy. Stem Cells Transl Med. (2014) 3(11):1363–74. 10.5966/sctm.2014-0017 25246698 PMC4214839

[36] Liu N Tang M Ding J. The interaction between nanoparticles-protein corona complex and cells and its toxic effect on cells. Chemosphere. (2020) 245:125624. 10.1016/j.chemosphere.2019.125624 31864050

[37] Sammons RL Haj AJE Marquis PM. Novel culture procedure permitting the synthesis of proteins by rat calvarial cells cultured on hydroxyapatite particles to be quantified. Biomat. (1994) 15(7):536–42. 10.1016/0142-9612(94)90020-5 7522593

[38] Pustylnikov S Sagar D Jain P Khan ZK. Targeting the C-type lectins-mediated host-pathogen interactions with dextran. J Pharm Pharm Sci. (2014) 17(3):371–92. 10.18433/J3N590 25224349 PMC5553543

[39] Chao Y Karmali PP Simberg D. Role of carbohydrate receptors in the macrophage uptake of dextran-coated iron oxide nanoparticles. In: Zahavy E Ordentlich A Yitzhaki S Shafferman A, editors. Nano-Biotechnology for biomedical and diagnostic research. Dordrecht: Springer Netherlands; 2012. p. 115–23. 10.1007/978-94-007-2555-3_11 22101717

[40] Bryan NS Grisham MB. Methods to detect nitric oxide and its metabolites in biological samples. Free Rad Biol and Med. (2007) 43(5):645–57. 10.1016/j.freeradbiomed.2007.04.026 17664129 PMC2041919

[41] Robertson BE Schubert R Hescheler J Nelson MT. cGMP-dependent protein kinase activates ca-activated K channels in cerebral artery smooth muscle cells. Am J Physiol-Cell Physiol. (1993) 265(1):C299–303. 10.1152/ajpcell.1993.265.1.C299 8338137

[42] Koh SD Monaghan K Sergeant GP Ro S Walker RL Sanders KM TREK-1 regulation by nitric oxide and cGMP-dependent protein kinase. An essential role in smooth muscle inhibitory neurotransmission. J Biol Chem. (2001) 276(47):44338–46. 10.1074/jbc.M108125200 11560940

[43] Feron O Saldana F Michel JB Michel T. The endothelial nitric-oxide synthase-caveolin regulatory cycle *. J Biol Chem. (1998) 273(6):3125–8. 10.1074/jbc.273.6.3125 9452418

[44] Ungvari Z Sun D Huang A Kaley G Koller A. Role of endothelial [Ca2+]i in activation of eNOS in pressurized arterioles by agonists and wall shear stress. Am J Physiol Heart Circ Physiol. (2001) 281(2):H606–H12. 10.1152/ajpheart.2001.281.2.H606 11454563

[45] Schneider J-C Kebir DE Chéreau C Lanone S Huang X-L Roessingh ASDB Involvement of Ca2+/calmodulin-dependent protein kinase II in endothelial NO production and endothelium-dependent relaxation. Am J Physiol Heart Circ Physiol. (2003) 284(6):H2311–H9. 10.1152/ajpheart.00932.2001 12560211

[46] Bongaerts M Aizel K Secret E Jan A Nahar T Raudzus F Parallelized manipulation of adherent living cells by magnetic nanoparticles-mediated forces. Int J Mol Sci. (2020) 21(18):6560. 10.3390/ijms21186560 32911745 PMC7555211

[47] Dhillon K Aizel K Broomhall TJ Secret E Goodman T Rotherham M Directional control of neurite outgrowth: emerging technologies for Parkinson's Disease using magnetic nanoparticles and magnetic field gradients. J R Soc Interface. (2022) 19(196):20220576. 10.1098/rsif.2022.0576 36349444 PMC9653228

