# Supplementary material for: Magnetic activation of TREK1 triggers stress signalling and regulates neuronal branching in SH-SY5Y cells
Source: Front Med Technol. 2022 Dec 5;4:981421. doi: 10.3389/fmedt.2022.981421 (PMC9761330; doi:10.3389/fmedt.2022.981421)
Supplement: Supplementary file 1 [file Datasheet1.docx]

Supplementary Material


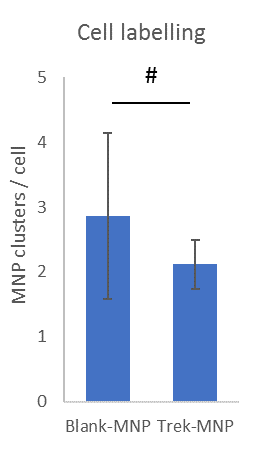


Figure S1: **Quantification of cell labelling**. Labelling of cells with control (Blank-MNP) resulted in an average of 2.9 MNP clusters/cell, labelling with TREK-MNP resulted in an average of 2.1 MNP clusters per cell. N=3, error bars represent standard deviation, # represents p>0.05.


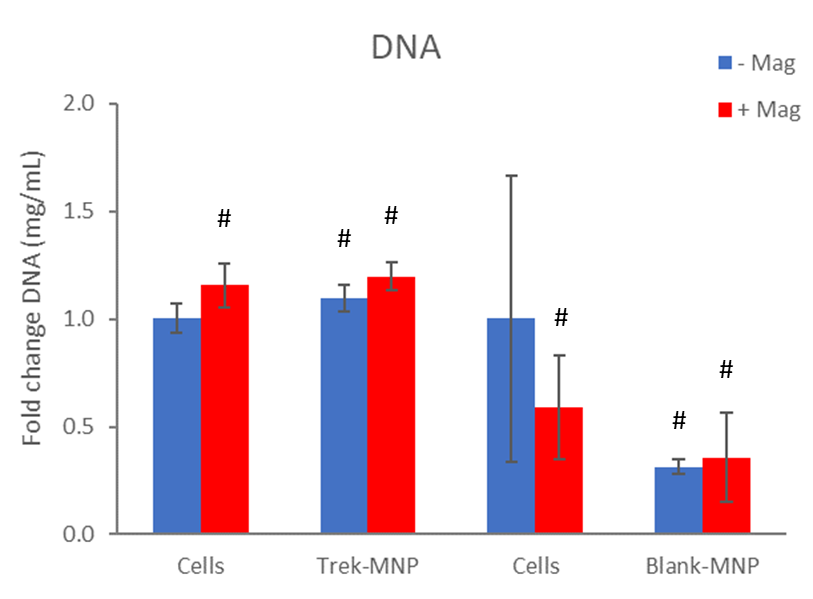


Figure S2: **MNP and magnetic field stimulation do not affect cell numbers**. The DNA content of cells was measured 24h after MNP treatment with or without magnetic field, these values were also used for Nitrite normalisation. DNA concentrations remained broadly consistent after treatment with either Trek-MNP or Blank-MNP, with or without magnetic field stimulation. n=3-4, bars represent mean fold change, # represents p>0.05 (2-way ANOVA).


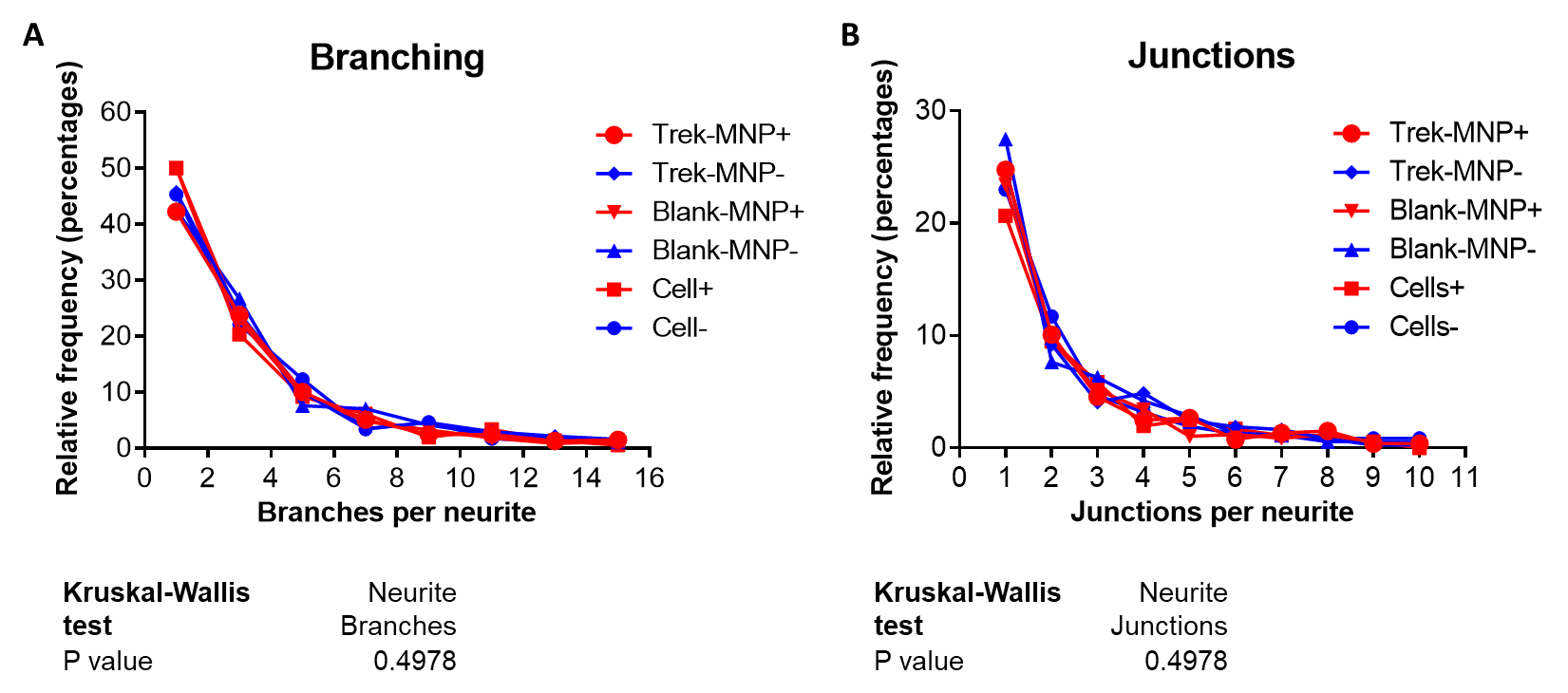


Figure S3: Neurite analysis of Immunofluorescent images indicated that neither TREK-MNP nor Control (Blank)-MNP with or without magnetic field stimulation affected the number of neurite branches (A) or Junctions (B). – represents treatments without magnetic field, + represents treatments with magnetic field stimulation. Histograms represent median value. Differences between groups were compared using non-parametric Kruskal-Wallis test. A minimum of 271 cells were analysed for each group.
